# Effect of time of day and seasonal variation on bronchodilator responsiveness: the SPIRO-TIMETRY study

**DOI:** 10.1136/thorax-2024-222773

**Published:** 2025-03-11

**Authors:** Ben Knox-Brown, Fu Chuen Kon, Karl Peter Sylvester, Akhilesh Jha

**Affiliations:** 1 Royal Papworth Hospital NHS Foundation Trust, Cambridge, UK; 2 Cambridge University Hospitals NHS Foundation Trust, Cambridge, UK; 3 Imperial College London National Heart and Lung Institute, London, UK; 4 Medicine, University of Cambridge, Cambridge, UK

**Keywords:** Asthma, Lung Physiology, Respiratory Measurement

## Abstract

We investigated the association between time of day and season of testing on the level of bronchodilator responsiveness in a hospital-based population. We found that per 1-hour increment in the working day, the odds of a positive bronchodilator response decreased by 8%. A similar effect was seen when time of day was dichotomised into morning and afternoon time periods. When stratifying by referral reason, the impact of time of day was only seen in those referred for asthma/query asthma. We also found that bronchodilator responsiveness was more common in winter months compared with the rest of the year.

## Introduction

 A key diagnostic criterion of asthma is bronchodilator responsiveness, traditionally defined as an improvement in the forced expiratory volume in 1 s (FEV_1_) or forced vital capacity (FVC) after bronchodilator administration.^1 2^

Diurnal variability in symptom presentation is characteristic of asthma, with previous research suggesting that a biological clock mechanism is an important factor in asthma pathogenesis.^3^ With respect to bronchodilator responsiveness, it is unknown if the effect of diurnal variability is restricted to only patients with asthma. Furthermore, the role of seasonal variation on bronchial hyperreactivity is unknown. We investigated the association between time of day and season of testing on the level of bronchodilator responsiveness.

## Methods

We retrospectively analysed data from patients referred to Cambridge University Hospitals NHS Foundation Trust between 1 January 2016 and 31 December 2023. Data were included in this study for all patients who were 18 years or older at the time of testing, who had acceptable and reproducible prebronchodilator and postbronchodilator spirometry.^4^ Data were extracted directly from the testing software.

Spirometry was instructed by certified physiologists. Spirometry was performed on the MasterScreen-PFT-Pro from Vyaire Medical before, and 20 min after inhalation of 400 µg of salbutamol delivered via a metered dose inhaler and spacer. Spirometers were calibrated daily and serviced according to manufacturer guidance. GLI race-neutral reference equations were used to calculate the lower limit of normal and percent predicted values.^5^ We defined bronchodilator responsiveness for both the American Thoracic Society/European Respiratory Society (ATS/ERS) 2005 (change in FEV_1_ or FVC≥12% and ≥200 mL of the initial value),^1^ and ATS/ERS 2022 definitions (change of >10% relative to the predicted value for FEV_1_ or FVC).^2^

We performed multivariable logistic regression analyses to investigate the influence of time of day on the odds of having a positive bronchodilator response. We considered time of day in the context of the working hours of the lung function testing laboratory (08:30–16:30). We modelled time as a continuous (per 1-hour increment), binary (08:30–13:30 vs 13:30–16:30) and categorical predictor (08:30–11:00 vs 11:01–14:00 vs 14:00–16:30). We adjusted regression models for age (years), sex (male/female), body mass index (BMI) (kg/m^2^), smoking history (ever/never) and prebronchodilator FEV_1_/FVC. To check for effect modification, we performed stratified analyses on those referred for asthma/query asthma vs any other referral reason. We also investigated the significance of any interaction between referral reason and time of day on the odds of having a positive bronchodilator response. We compared the results from the model with the interaction term to a model with the individual predictors using the likelihood ratio test. Referral reason was based on clinical judgement and extracted from the consultant referral document. We repeated the above analyses to investigate the influence of seasonal variation on bronchodilator responsiveness. We considered results significant if the p value was less than 0.05. Analyses were performed using Stata V.18 (StataCorp).

## Results

**Figure 1:**
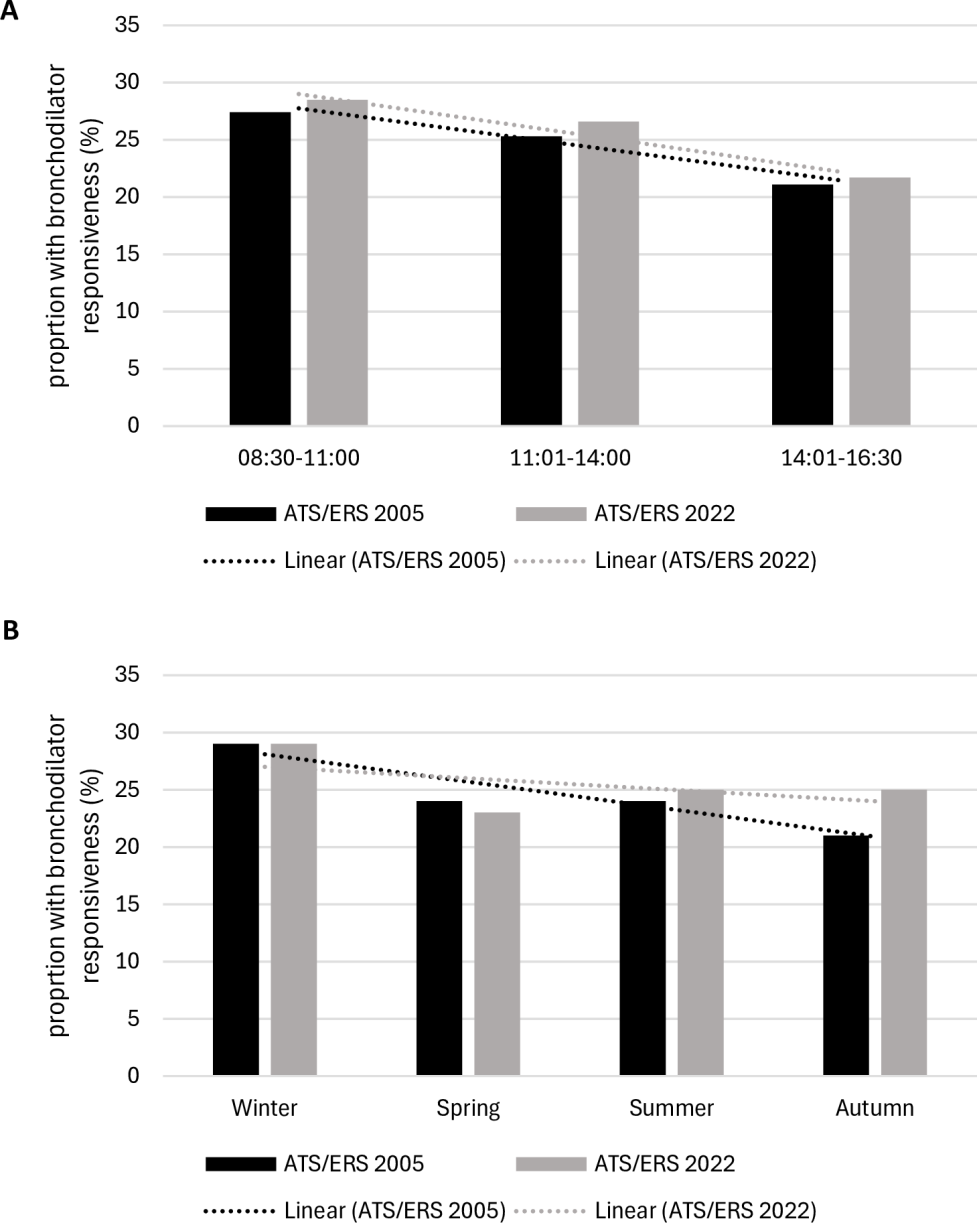
Graph showing (A) the association between time of test and bronchodilator responsiveness and (B) the association between season of test and bronchodilator responsiveness. ATS/ERS, American Thoracic Society/European Respiratory Society.

Data from 1620 individual patients were included in our analyses (online supplemental efigure 1). 62% (1004 of 1620) were female, mean (SD) age was 53.2 (16.1) years and mean BMI was 29.4 kg/m^2^ (7.1). 48% (722 of 1620) of patients reported having ever smoked, with 58% (944 of 1620) referred for asthma/query asthma. Other referral reasons are described in online supplemental etable 1. 25% of patients (400 of 1620) had bronchodilator responsiveness according to the ATS/ERS 2005 definition, compared with 26% (416 of 1620) of patients when using the 2022 definition. A greater proportion of patients tested in the morning had bronchodilator responsiveness compared with those tested in the afternoon for both ATS/ERS 2005 (28% vs 22%) and ATS/ERS 2022 (28% vs 23%) definitions (table 1, figure 1a).

**Table 1:** Characteristics of study participants overall and by testing time

	Overall(n=1620)	Morning08:30–12:30(n=852)	Afternoon13:30–16:30(n=768)	P value
Demographics				
Age, years, mean (SD)	53.2 (16.1)	53.4 (15.9)	53.1 (16.5)	0.704
Female, n (%)	1004 (62%)	540 (63%)	464 (60%)	0.220
White, n %	1498 (92%)	784 (92%)	714 (93%)	0.469
BMI, kg/m^2^, mean (SD)	29.4 (7.1)	29.8 (7.6)	28.9 (6.4)	0.006
Ever smoked, n (%)	772 (48%)	407 (48%)	365 (48%)	0.922
Referred for asthma/query asthma, n (%)	944 (58%)	523 (61%)	421 (55%)	0.007
Baseline spirometry				
FEV_1_, L, mean (SD)	2.5 (1.0)	2.4 (0.9)	2.6 (1.0)	0.001
FEV_1_, pp, mean (SD)	86.5 (23.7)	84.3 (24.1)	88.9 (23.1)	<0.001
FVC, L, mean (SD)	3.7 (1.2)	3.6 (1.4)	3.7 (1.2)	0.017
FVC, pp, mean (SD)	101.0 (20.0)	99.5 (20.2)	102.7 (19.6)	0.001
FEV_1_/FVC, %, mean (SD)	52.3 (23.4)	54.4 (22.8)	49.9 (23.9)	<0.001
FEV_1_/FVC<LLN, n (%)	1047 (65%)	530 (62%)	517 (67%)	0.032
Postbronchodilator spirometry				
FEV_1_, L, mean (SD)	2.7 (1.0)	2.6 (0.9)	2.8 (1.0)	0.006
FEV_1_ % change, median (IQR)	5.7 (1.9, 11.4)	6.5 (2.1, 12.6)	5.3 (1.9, 10.6)	0.010
FVC, L, mean (SD)	3.7 (1.2)	3.7 (1.1)	3.8 (1.2)	0.010
FVC % change, median (IQR)	1.0 (−1.7, 5.2)	1.4 (−1.6, 5.9)	0.5 (−2.0, 4.4)	0.002
FEV_1_/FVC, %, mean (SD)	53.9 (25.4)	56.6 (24.2)	50.9 (26.0)	<0.001
FEV_1_/FVC<LLN, n (%)	933 (58%)	467 (55%)	466 (61%)	0.017
BDR 2005 definition, n (%)	400 (25%)	234 (28%)	166 (22%)	0.006
BDR 2022 definition, n (%)	416 (26%)	242 (28%)	174 (23%)	0.008

Continuous variables are reported as mean with SD or median with IQR. Categorical variables are reported as number (n) with percentage (%). Independent t-test is used to compare differences in means, and Mann-Whitney U test is used to compare differences in medians between morning versus afternoon testing. χ2 test is used to compare differences in the number and percentage in each category between morning versus afternoon testing. A p<0.05 is considered significant.

ATS/ERS 2005 definition: change in FEV_1_ or FVC ≥12% and ≥200mL of the initial value.^1^

Reversibility ATS/ERS 2022 definition: change of >10% relative to the predicted value for FEV_1_ or FVC.^2^

Predicted values for FEV_1_ and FVC and the LLN for FEV_1_/FVC calculated using race-neutral reference equations from the Global Lung Initiative.^5^

ATS/ERS, American Thoracic Society/European Respiratory Society; BDR, bronchodilator responsiveness; BMI, body mass index; % change, per cent change pre to post bronchodilator; FEV_1_, forced expiratory volume in 1 s; FVC, forced vital capacity; LLN, lower limit of normal; pp, per cent predicted.

The results of the multivariable logistic regression showed that per 1-hour increment in the working day, the odds of having bronchodilator responsiveness declined by 8% (OR 0.92, 95% CI 0.88 to 0.97). When the time of day was dichotomised into morning and afternoon sessions, there was a similar association, with reduced odds of bronchodilator responsiveness in the afternoon (OR 0.68, 95% CI 0.54 to 0.85) (table 2). Patients referred for asthma/query asthma had a significant association with bronchodilator responsiveness, with the magnitude of the effect similar to that of the main analysis, while there was no association in those referred for other reasons (online supplemental etables 2,3). There was also evidence of a significant interaction between referral reason and time of day (per 1-hour increment) on the odds of having a positive bronchodilator response (p value for likelihood ratio test <0.001).

**Table 2:** Logistic regression analysis of the association between time of test and having bronchodilator responsiveness

	Total n	ATS/ERS BDR 2005			ATS/ERS BDR 2022		
		n (%)	OR (95%CI)	P value	n (%)	OR (95%CI)	P value
Time (per hour increment)	1620	400 (25%)	0.92 (0.88, 0.97)	0.001	416 (26%)	0.92 (0.88, 0.97)	0.001
Time (binary)							
08:30–12:30	852	234 (28%)	ref	ref	242 (28%)	ref	ref
13:30–16:30	768	166 (22%)	0.66 (0.52, 0.83)	0.001	174 (23%)	0.68 (0.54, 0.85)	0.001
Time (tertiles)							
08:30–11:00	547	150 (27%)	ref	ref	156 (29%)	ref	ref
11:01–14:00	552	140 (25%)	0.86 (0.66, 1.14)	0.316	147 (27%)	0.88 (0.67, 0.16)	0.373
14:01–16:30	521	110 (21%)	0.61 (0.45, 0.81)	0.001	113 (22%)	0.61 (0.46, 0.81)	0.001

Models adjusted for age, BMI, sex, baseline FEV_1_/FVC and smoking status.

A p<0.05 is considered significant.

ATS/ERS 2005 definition: change in FEV_1_ or FVC ≥12% and ≥200mL of the initial value.^1^

Bronchodilator responsiveness ATS/ERS 2022 definition: change of >10% relative to the predicted value for FEV_1_ or FVC.^2^

Predicted values for FEV_1_ and FVC and the LLN for FEV_1_/FVC are calculated using race-neutral reference equations from the Global Lung Initiative.^5^

ATS/ERS, American Thoracic Society/European Respiratory Society; BDR, bronchodilator responsiveness; FEV_1_, forced expiratory volume in 1 s; FVC, forced vital capacity; LLN, lower limit of normal.

For the seasonal analysis, proportionally more patients tested in the winter had bronchodilator responsiveness compared with other seasons (figure 1b). Compared with those tested in winter, patients tested in autumn had significantly lower odds of having bronchodilator responsiveness but only for those with bronchodilator responsiveness according to the ATS/ERS 2005 definition (OR 0.67, 95% CI 0.48 to 0.92) (online supplemental etables 4,5)

## Discussion

We have found that in contrast to patients tested in the afternoon, patients tested for asthma/query asthma in the morning had lower lung function and were more likely to exhibit bronchodilator responsiveness. Studies have shown that the clock protein REV-ERBα controls airway hyperresponsiveness at different times of day, and that knocking down the *Rev-erbα* gene abolishes the time-of-day effect.^6 7^ The results from our study are consistent with the recently published RADicA study, which in a relatively small sample of patients with suspected asthma found that patients self-reported their worst symptoms in the morning and night, and that patients were more likely to demonstrate bronchodilator responsiveness in the morning compared with the afternoon.^8^ Taken together, these findings suggest that performing diagnostic testing when patients are most symptomatic, that is, in the morning, may improve diagnostic testing. This also reinforces the need to document the timing of spirometry testing whenever possible, and that repeat testing should be done at the same time of day.

Similarly, we also found seasonal variation in bronchodilator responsiveness, with bronchodilator responsiveness more likely to be seen in the winter months. Interestingly, previous groups found that adults or children with asthma or allergic rhinitis display greater FeNO and methacholine hyperresponsiveness during pollen season, characteristically seen during summer.^9 10^ We saw a slight increase in the proportion with bronchodilator responsiveness in the summer compared with the spring and autumn, but still to a lesser extent than seen in the winter. This could be because previous research has focused on a younger, more atopic population where an IgE response is more predominant, compared with older adults where asthma mortality and hospital admissions are higher during winter.

Although limited by cross-sectional study design and a lack of information regarding the formal diagnosis, we show that patients with asthma/query asthma are more likely to demonstrate bronchodilator responsiveness if spirometry testing is done in the morning compared with later in the working day. We also show that bronchodilator responsiveness is more likely in the winter months. This should be considered by clinicians when interpreting the results of bronchodilator responsiveness testing. It is important that prospective studies are conducted to confirm our findings, with particular focus on repeated measurements of the same individuals at different times of the day.

## Supplementary material

10.1136/thorax-2024-222773 online supplemental file 1

10.1136/thorax-2024-222773 online supplemental file 2

## References

[1] Miller MR, Hankinson J, Brusasco V (2005). Standardisation of spirometry. Eur Respir J.

[2] Stanojevic S, Kaminsky DA, Miller MR (2022). ERS/ATS technical standard on interpretive strategies for routine lung function tests. Eur Respir J.

[3] Wang R, Murray CS, Fowler SJ (2021). Asthma diagnosis: into the fourth dimension. Thorax.

[4] Graham BL, Steenbruggen I, Miller MR (2019). Standardization of Spirometry 2019 Update. An Official American Thoracic Society and European Respiratory Society Technical Statement. Am J Respir Crit Care Med.

[5] Bowerman C, Bhakta NR, Brazzale D (2023). A Race-neutral Approach to the Interpretation of Lung Function Measurements. Am J Respir Crit Care Med.

[6] Scheer FAJL, Hilton MF, Evoniuk HL (2021). The endogenous circadian system worsens asthma at night independent of sleep and other daily behavioral or environmental cycles. Proc Natl Acad Sci U S A.

[7] Durrington HJ, Krakowiak K, Meijer P (2020). Circadian asthma airway responses are gated by REV-ERBα. Eur Respir J.

[8] Wang R, Fowler SJ, Maidstone R (2024). The impact of time of day on the diagnostic performance of tests for asthma. ERJ Open Res.

[9] Vahlkvist S, Sinding M, Skamstrup K (2006). Daily home measurements of exhaled nitric oxide in asthmatic children during natural birch pollen exposure. J Allergy Clin Immunol.

[10] Skiepko R, Zietkowski Z, Tomasiak-Lozowska MM (2011). Bronchial hyperresponsiveness and airway inflammation in patients with seasonal allergic rhinitis. J Investig Allergol Clin Immunol.

