# Supplementary material for: Effect of time of day and seasonal variation on bronchodilator responsiveness: the SPIRO-TIMETRY study
Source: Thorax. 2025 Mar 11;80(6):e222773. doi: 10.1136/thorax-2024-222773 (PMC12128765; doi:10.1136/thorax-2024-222773)
Supplement: online supplemental file 1 [file thorax-80-6-s001.pdf]

Supplemental material

Contents

eFigure 1. Study flow diagram 2

eTable 1. List of referral reasons for those not defined as asthma/query asthma 3

eTable 2. Characteristics of study participants referred for asthma/query asthma vs other 4

eTable 3. Logistic regression analysis of the association between time of test and having bronchodilator responsiveness in those referred for asthma/query asthma compared to other referral reasons 5

eTable 4. Characteristics of study participants by season tested 7

eTable 5. Logistic regression analysis of the association between the season of test and having bronchodilator responsiveness 8

References 9

eFigure 1. Study flow diagram

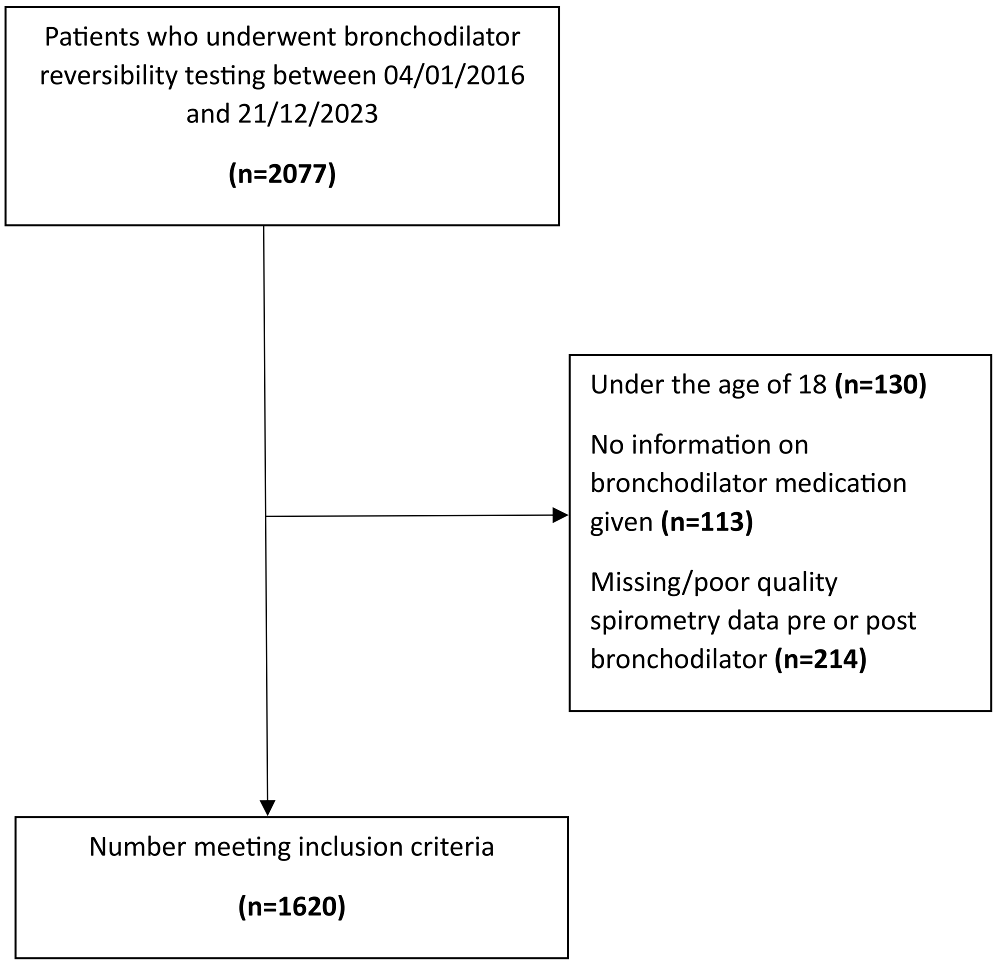

eTable 1. List of referral reasons for those not defined as asthma/query asthma

	**N=676**
COPD/query COPD, n (%)	73 (11%)
Shortness of breath of unknown cause, n (%)	339 (50%)
Bronchiectasis, n (%)	44 (7%)
Chronic cough, n (%)	148 (22%)
Vasculitis, n (%)	29 (4%)
Rheumatoid lung disease, n (%)	21 (3%)
Interstitial lung disease, n (%)	22 (3%)

eTable 2. Characteristics of study participants referred for asthma/query asthma vs other

	Other (n=676)	Asthma/query asthma (n=944)	P-value
**Demographics**			
Age, years, mean (SD)	56.8 (15.3)	50.7 (16.3)	<0.001
Female, n (%)	380 (56%)	624 (66%)	<0.001
White, n %	628 (93%)	870 (92%)	0.579
BMI, kg/m^2, mean (SD)	28.4 (6.0)	30.1 (7.7)	<0.001
Ever smoked, n (%)	369 (55%)	403 (43%)	<0.001
08:30-12:30, n (%)	329 (49%)	523 (55%)	0.007
**Baseline spirometry**			
FEV_1, L, mean (SD)	2.5 (1.0)	2.5 (0.9)	0.655
FEV_1, pp, mean (SD)	87.9 (24.4)	85.5 (23.1)	0.046
FVC, L, mean (SD)	3.7 (1.2)	3.6 (1.1)	0.769
FVC, pp, mean (SD)	101.5 (20.5)	100.6 (19.6)	0.407
FEV_1/FVC, %, mean (SD)	55.7 (23.1)	49.9 (23.3)	<0.001
FEV_1/FVC <LLN, n (%)	389 (58%)	658 (70%)	<0.001
**Post-bronchodilator spirometry**			
FEV_1, L, mean (SD)	2.7 (1.0)	2.7 (0.9)	0.274
FEV_1 % change, median (IQR)	4.5 (1.3, 9.5)	6.9 (2.5, 14.0)	<0.001
FVC, L, mean (SD)	2.7 (1.2)	3.7 (1.1)	0.316
FVC % change, median (IQR)	0.1 (-2.4, 4.1)	1.5 (-1.2, 6.3)	<0.001
FEV_1/FVC, %, mean (SD)	57.4 (24.7)	51.4 (25.3)	<0.001
FEV_1/FVC <LLN, n (%)	341 (50%)	592 (62%)	<0.001
**BDR 2005 definition, n (%)**	108 (16%)	292 (31%)	<0.001
**BDR 2022 definition, n (%)**	112 (17%)	304 (32%)	<0.001

Continuous variables reported as mean with standard deviation (SD) or median with interquartile range (IQR). Categorical variables reported as number (n) with percentage (%). Independent t-test used to compare differences in means and Mann-Whitney U test used to compare differences in medians between other vs asthma/query asthma. Chi-squared test used to compare differences in the number and percentage in each category between other vs asthma/query asthma. P<0.05 considered significant. "Other" referral reasons broadly include COPD/A1ATD, vasculitis, sarcoidosis, bronchiectasis, unknown shortness of breath, cough, rheumatoid lung disease, interstitial lung disease, pre-operative assessment, and immunodeficiency. BMI: Body mass index; FEV_1: Forced expiratory volume in 1 second; L: Litres; FVC: Forced vital capacity; LLN: Lower limit of normal; pp: percent predicted; % change: percent change pre to post bronchodilator; BDR: Bronchodilator responsiveness; ATS/ERS: American Thoracic Society/European Respiratory Society. ATS/ERS 2005 definition: change in FEV_1 or FVC ≥12% and ≥200mL of the initial value (1); Reversibility ATS/ERS 2022 definition: change of >10% relative to the predicted value for FEV_1 or FVC (2). Predicted values for FEV_1 and FVC and the LLN for FEV_1/FVC calculated using race neutral reference equations from the Global Lung Initiative (3).

eTable 3. Logistic regression analysis of the association between time of test and having bronchodilator responsiveness in those referred for asthma/query asthma compared to other referral reasons

		Total	ATS/ERS BDR 2005			ATS/ERS BDR 2022		
		n	*n* (%)	OR (95%CI)	P-value	*n* (%)	OR (95%CI)	P-value
**Asthma/query asthma**	**Time (per hour increment)**	944	292 (31%)	0.92 (0.87, 0.98)	0.008	304 (32%)	0.94 (0.88,0.99)	0.028
	**Time (binary)**							
	08:30-12:30	523	*176 (33%)*	*ref*	*ref*	*180 (34%)*	*ref*	*ref*
	13:30-16:30	421	116 (27%)	0.69 (0.52, 0.93)	0.014	124 (29%)	0.75 (0.56, 0.99)	0.043
	**Time (tertiles)**							
	08:30-11:00	335	*113 (34%)*	*ref*	*ref*	*115 (34%)*	*ref*	*ref*
	11:01-14:00	319	106 (33%)	0.96 (0.69, 1.34)	0.830	111 (35%)	1.01 (0.73, 0.40)	0.948
	14:01-16:30	290	73 (25%)	0.58 (0.40, 0.83)	0.003	78 (29%)	0.63 (0.44, 0.90)	0.011
**Other**	**Time (per hour increment)**	676	108 (16%)	0.95 (0.87, 1.04)	0.280	112 (17%)	0.92 (0.84, 1.01)	0.093
	**Time (binary)**							
	08:30-12:30	329	58 (18%)	*ref*	*ref*	62 (19%)	*ref*	*ref*
	13:30-16:30	347	50 (14%)	0.70 (0.46, 1.08)	0.114	50 (14%)	0.65 (0.42, 1.00)	0.046
	**Time (tertiles)**							
	08:30-11:00	212	37 (17%)	*ref*	*ref*	41 (19%)	*ref*	*ref*
	11:01-14:00	233	34 (15%)	0.78 (0.46, 1.33)	0.373	36 (15%)	0.74 (0.44, 1.23)	0.240
	14:01-16:30	231	37 (16%)	0.81 (0.48, 1.36)	0.425	35 (15%)	0.66 (0.40. 1.12)	0.122

Models Adjusted for Age, BMI, Sex, baseline FEV_1/FVC, and smoking status. $P<0.05$ considered significant. OR: Odds ratio; CI: Confidence interval; ATS/ERS: American Thoracic Society/European Respiratory Society; BDR: Bronchodilator responsiveness; ATS/ERS 2005 definition: change in FEV_1 or FVC ≥12% and ≥200mL of the initial value (1); Reversibility ATS/ERS 2022 definition: change of >10% relative to the predicted value for FEV_1 or FVC (2). Predicted values for FEV_1 and FVC and the LLN for FEV_1/FVC calculated using race neutral reference equations from the Global Lung

Initiative (3). “Other” referral reasons broadly include COPD/A1ATD, vasculitis, sarcoidosis, bronchiectasis, unknown shortness of breath, cough, rheumatoid lung disease, interstitial lung disease, pre-operative assessment, and immunodeficiency

eTable 4. Characteristics of study participants by season tested

	Winter (n=390)	Spring (n=353)	Summer (n=451)	Autumn (n=456)	P-value
**Demographics**					
Age, years, mean (SD)	52.5 (16.7)	53.7 (16.4)	54.7 (16.4)	52.4 (15.8)	0.256
Female, n (%)	237 (61%)	222 (63%)	300 (67%)	245 (58%)	0.048
BMI, kg/m^2, mean (SD)	29.4 (7.2)	28.8 (6.0)	30.0 (7.9)	29.1 (6.8)	0.131
Ever smoked, n (%)	203 (52%)	168 (48%)	209 (46%)	192 (45%)	0.215
Referred for asthma/query asthma, n (%)	232 (59%)	193 (55%)	262 (58%)	257 (60%)	0.413
**Baseline spirometry**					
FEV_1, L, mean (SD)	2.5 (1.0)	2.5 (1.0)	2.4 (1.0)	2.6 (0.9)	0.147
FEV_1, pp, mean (SD)	85.2 (24.0)	86.1 (24.0)	86.4 (23.6)	88.1 (23.2)	0.367
FVC, L, mean (SD)	3.7 (1.2)	3.7 (1.2)	3.5 (1.1)	3.7 (1.1)	0.096
FVC, pp, mean (SD)	100.9 (19.8)	102.6 (19.0)	100.4 (20.2)	100.3 (20.7)	0.371
FEV_1/FVC, %, mean (SD)	51.3 (23.2)	53.7 (23.0)	51.8 (23.7)	52.6 (23.6)	0.5116
FEV_1/FVC <LLN, n (%)	267 (68%)	218 (61%)	288 (64%)	274 (64%)	0.271
**Post-bronchodilator spirometry**					
FEV_1, L, mean (SD)	2.7 (1.0)	2.7 (1.0)	2.6 (1.0)	2.8 (1.0)	0.194
FEV_1 % change, median (IQR)	6.3 (2.2, 13.2)	6.1 (1.9, 11.7)	5.5 (2.1, 11.9)	5.2 (1.7, 10.7)	0.120
FVC, L, mean (SD)	3.8 (1.2)	3.8 (1.2)	3.6 (1.1)	3.7 (1.2)	0.064
FVC % change, median (IQR)	1.2 (-1.3, 6.4)	1.0 (-1.5, 4.6)	0.9 (-1.9, 5.0)	0.7 (-2.1, 4.7)	0.649
FEV_1/FVC, %, mean (SD)	52.7 (25.2)	55.6 (24.8)	53.7 (23.4)	53.9 (25.5)	0.477
FEV_1/FVC <LLN, n (%)	243 (62%)	197 (56%)	251 (56%)	242 (57%)	0.186
**BDR 2005 definition, n (%)**	115 (29%)	85 (24%)	109 (24%)	91 (21%)	0.048
**BDR 2021 definition, n (%)**	113 (29%)	83 (23%)	114 (25%)	106 (25%)	0.352

Continuous variables reported as mean with standard deviation (SD) or median with interquartile range (IQR). Categorical variables reported as number (n) with percentage (%). ANOVA used to compare differences in means between seasons for normally distributed variables. Friedmans test used to compare differences between medians. Chi-squared test used to compare differences in the number and percentage in each category between seasons. $P<0.05$ considered significant. BMI: Body mass index; FEV_1: Forced expiratory volume in 1 second; L: Litres; FVC: Forced vital capacity; LLN: Lower limit of normal; pp: percent predicted; % change: percent change pre to post bronchodilator; BDR: Bronchodilator responsiveness; ATS/ERS: American Thoracic Society/European Respiratory Society. ATS/ERS 2005 definition: change in FEV_1 or FVC ≥12% and ≥200mL of the initial value (1); Reversibility ATS/ERS 2022 definition: change of >10% relative to the predicted value for FEV_1 or FVC (2). Predicted values for FEV_1 and FVC and the LLN for FEV_1/FVC calculated using race neutral reference equations from the Global Lung Initiative (3).

eTable 5. Logistic regression analysis of the association between the season of test and having bronchodilator responsiveness

Season	Total	ATS/ERS BDR 2005			ATS/ERS BDR 2022		
	n	***n*** **(%)**	**OR (95%CI)**	**P-value**	***n*** **(%)**	**OR (95%CI)**	**P-value**
Winter	390	115 (29%)	*ref*	*ref*	113 (29%)	*ref*	*ref*
Spring	353	85 (24%)	0.81 (0.58, 1.14)	0.227	83 (23%)	0.79 (0.57, 1.11)	0.182
Summer	451	109 (24%)	0.76 (0.55, 1.04)	0.093	114 (26%)	0.83 (0.61, 1.14)	0.245
Autumn	426	91 (21%)	0.67 (0.48, 0.92)	0.015	106 (25%)	0.84 (0.62, 1.16)	0.302

Models Adjusted for Age, BMI, Sex, baseline FEV_1/FVC, and smoking status. P<0.05 considered significant. OR: Odds ratio; CI: Confidence interval; ATS/ERS: American Thoracic Society/European Respiratory Society; BDR: Bronchodilator responsiveness; ATS/ERS 2005 definition: change in FEV_1 or FVC ≥12% and ≥200mL of the initial value (1); Reversibility ATS/ERS 2022 definition: change of >10% relative to the predicted value for FEV_1 or FVC (2). Predicted values for FEV_1 and FVC and the LLN for FEV_1/FVC calculated using race neutral reference equations from the Global Lung Initiative (3).

References

1. Miller MR, Hankinson J, Brusasco V, Burgos F, Casaburi R, Coates A, et al. Standardisation of spirometry. Eur Respir J. 2005;26(2):319-38.
2. Stanojevic S, Kaminsky DA, Miller MR, Thompson B, Aliverti A, Barjaktarevic I, et al. ERS/ATS technical standard on interpretive strategies for routine lung function tests. Eur Respir J. 2022;60(1).
3. Bowerman C, Bhakta NR, Brazzale D, Cooper BR, Cooper J, Gochicoa-Rangel L, et al. A Race-neutral Approach to the Interpretation of Lung Function Measurements. Am J Respir Crit Care Med. 2023;207(6):768-74.
