# Supplementary material for: Effect of time of day and seasonal variation on bronchodilator responsiveness: the SPIRO-TIMETRY study
Source: Thorax. 2025 Mar 11;80(6):e222773. doi: 10.1136/thorax-2024-222773 (PMC12128765; doi:10.1136/thorax-2024-222773)
Supplement: online supplemental file 2 [file thorax-80-6-s002.pptx]

## Slide 1
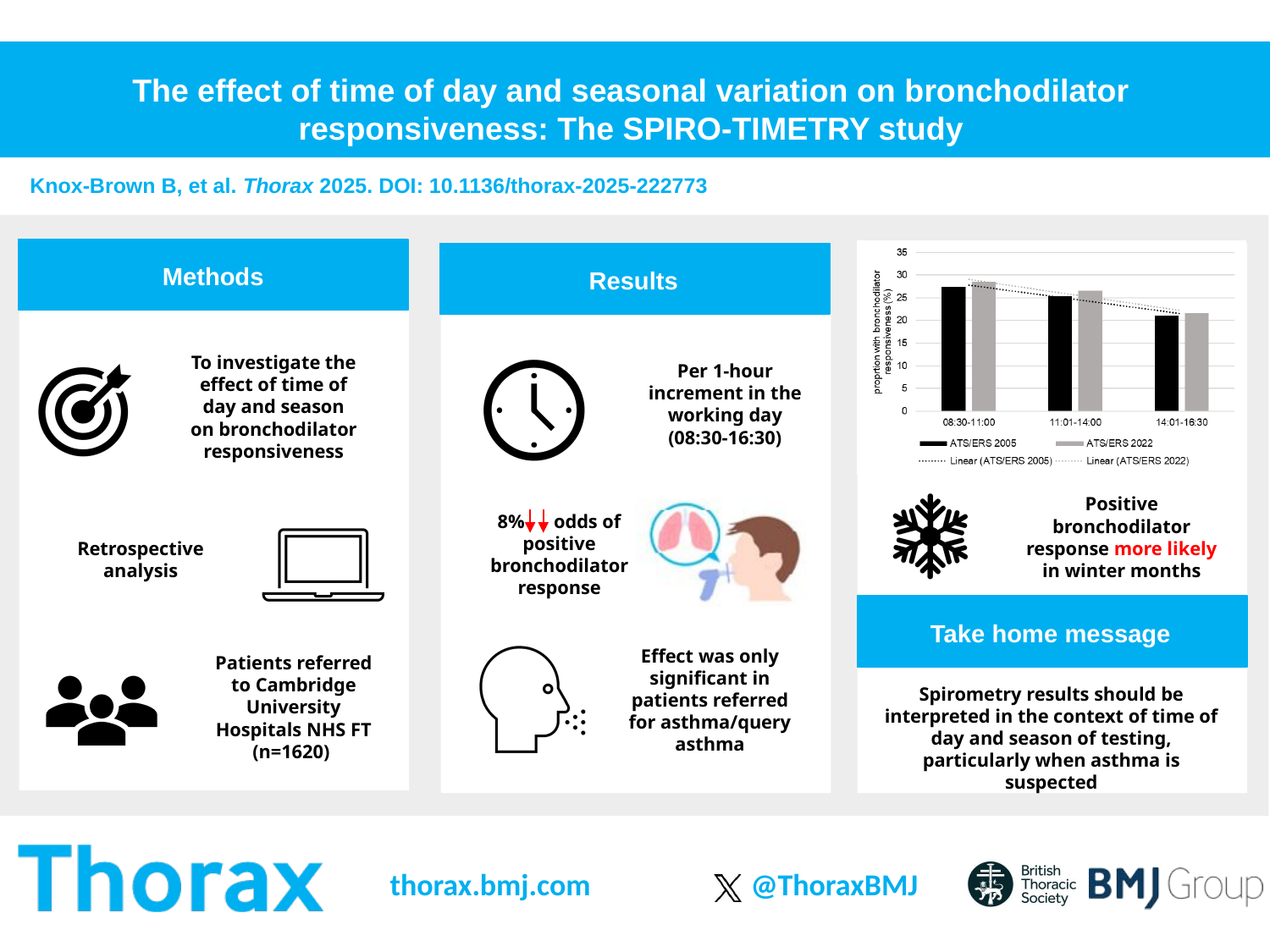

The effect of time of day and seasonal variation on bronchodilator responsiveness: The SPIRO-TIMETRY study
Knox-Brown B, et al. Thorax 2025. DOI: 10.1136/thorax-2025-222773
Methods
Results
Manuscript Title
To investigate the effect of time of day and season on bronchodilator responsiveness
Per 1-hour increment in the working day (08:30-16:30)
Positive bronchodilator response more likely in winter months
8% odds of positive bronchodilator response
Retrospective analysis
Take home message
Effect was only significant in patients referred for asthma/query asthma
Patients referred to Cambridge University Hospitals NHS FT (n=1620)
Spirometry results should be interpreted in the context of time of day and season of testing, particularly when asthma is suspected
© Author(s) (or their employer(s) 2019. Re-use permitted under CC BY. Published by BMJ.
thorax.bmj.com @ThoraxBMJ
